# Application of Monoclonal Antibody G250 Recognizing Carbonic Anhydrase IX in Renal Cell Carcinoma

**DOI:** 10.3390/ijms140611402

**Published:** 2013-05-29

**Authors:** Jeannette C. Oosterwijk-Wakka, Otto C. Boerman, Peter F. A. Mulders, Egbert Oosterwijk

**Affiliations:** 1 Department of Urology, Experimental Urology (267), University Medical Centre St. Radboud, P.O. Box 9101, Nijmegen 6500 HB, The Netherlands; E-Mail: e.oosterwijk@uro.umcn.nl; 2 Department of Nuclear Medicine (756), University Medical Centre St. Radboud, P.O. Box 9101, Nijmegen 6500 HB, The Netherlands; E-Mail: O.Boerman@nucmed.umcn.nl; 3 Department of Urology (659), University Medical Centre St. Radboud, P.O. Box 9101, Nijmegen 6500 HB, The Netherlands; E-Mail: P.Mulders@uro.umcn.nl

**Keywords:** CAIX, monoclonal antibody, G250, girentuximab, clear cell renal cell carcinoma, RCC, radioimmunotherapy, imaging

## Abstract

Monoclonal antibody G250 (mAbG250) recognizes a determinant on carbonic anhydrase IX (CAIX). CAIX is expressed by virtually all renal cell carcinomas of the clear cell type (ccRCC), but expression in normal tissues is restricted. The homogeneous CAIX expression in ccRCC and excellent targeting capability of mAbG250 in animal models led to the initiation of the clinical evaluation of mAbG250 in (metastatic) RCC (mRCC) patients. Clinical studies confirmed the outstanding targeting ability of mAbG250 and cG250 PET imaging, as diagnostic modality holds great promise for the future, both in detecting localized and advanced disease. Confirmation of the results obtained in the non-randomized clinical trials with unmodified cG250 is needed to substantiate the value of cG250 treatment in mRCC. cG250-Based radio immuno-therapy (RIT) holds promise for treatment of patients with small-volume disease, and adjuvant treatment with unmodified cG250 may be of value in selected cases. In the upcoming years, ongoing clinical trials should provide evidence for these assumptions. Lastly, whether cG250-based RIT can be combined with tyrosine kinase inhibitors, which constitutes the current standard treatment for mRCC, needs to be established.

## 1. Introduction

Renal cell carcinoma (RCC) accounts for approximately 3% of all cancers and was estimated to be diagnosed in over 60,000 individuals in the United States in 2011 [1]. The most prominent subtype of RCC (~70%) is clear cell (ccRCC). In approximately 70% of patients, the tumor is confined to the kidney at presentation. In 30% of cases, patients present with or develop metastases at a later time point. Patients with advanced disease have a poor prognosis with an overall five-year survival of <10%. Based on the molecular insight that ccRCC is characterized by molecular aberrations that leads to high expression levels of amongst others VEGF, various anti-angiogenic therapies have been developed. For patients with metastatic RCC (mRCC), several anti-angiogenic therapies are available [2–7] Implementation of these new treatment modalities has led to a significant increase in progression-free survival [8]. However, complete responses (CR) are rare, and long-lasting stable disease (SD) is often seen, but eventually all patients progress. Moreover, frequently significant toxicity can occur, which may lead to drug cessation or dose reduction.

Monoclonal antibody G250 (mAbG250) was isolated more than 25 years ago from a hybridoma produced from a splenocyte of a mouse immunized with a fresh human RCC [9]. Immunohistochemical analysis of renal tumors showed homogeneous expression in the vast majority (>80%) of primary RCC and about 70% of mRCC lesions. Analyses of non-RCC tumors revealed variable, non-homogeneous staining. Initial specificity analysis on normal human tissues revealed cross-reactivity with gastric mucosal cells and large bile ducts. Subsequent in-depth fine-specificity analysis revealed reactivity with epithelial cells of the upper gastrointestinal tract and pancreatic cells. Originally, no association with a particular histological RCC subtype was noted, but it is now clear that the antigen recognized by mAbG250 is almost ubiquitously expressed in ccRCC [10,11]. Based on this fine-specificity analysis, mAbG250 target antigen was readily suggested as a potential diagnostic and therapeutic target.

## 2. Cloning of G250 Antigen and Relation to ccRCC

The general occurrence of the antigen recognized by mAbG250 in RCC and absence from normal kidney suggested that the aberrant expression was inherently related to tumor development, possibly due to a common initiating event [9].

**Figure 1:**
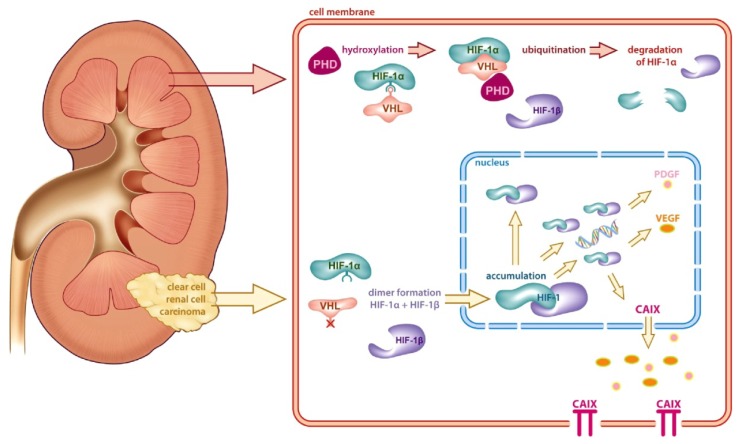
Schematic representation of regulation of carbonic anhydrase IX expression (CAIX) in kidney. In normal kidney tissue, hypoxia inducible factor-1α (HIF-1α) is hydroxylated by prolyl hydroxylase domain proteins (PHD) and bound by Von Hippel-Lindau protein (pVHL). Subsequently, the complex is ubiquitinated, which causes degradation of HIF-1α. In clear cell renal cell carcinoma (ccRCC), pVHL is mutated and binding with HIF-1α is prohibited. Subsequently HIF-1α forms a heterodimeric complex with HIF-1β, translocates to the nucleus, where it activates hypoxia inducible genes, such as vascular endothelial growth factor and CAIX, which is expressed on the tumor cell membrane. Reproduced with permission from Stillebroer *et al*., European Urology, published by Elsevier, July, 2010; 58(1): 75–83 [20].

Cloning of the antigen recognized by mAbG250 showed that mAbG250 recognized a conformational determinant of carbonic anhydrase IX (CAIX), a gene originally identified in HeLa cells [12,13]. CAIX is a member of the carbonic anhydrase group of enzymes, has a transmembrane, as well as a cytosolic domain, and catalyzes the reaction: CO_2_ + H_2_O↔HCO_3_^−^ + H^+^. Extensive molecular studies of the CAIX promoter region demonstrated that HIF-1α binding was an absolute requirement for CAIX expression in ccRCC [14]. This finding uncovered a direct molecular link between the observed ccRCC-specificity of mAbG250 and the molecular events leading to ccRCC. Elegant molecular studies in families suffering from Von Hippel-Lindau (VHL) syndrome, an autosomal dominant disease, showed that defects in the VHL gene were responsible for tumor development. These patients develop multiple tumors, including ccRCC. Studies in sporadic ccRCC demonstrated that also in these cases, VHL was mutated [15]. Subsequent studies showed that VHL is involved in the hypoxic response: under normoxic conditions, hypoxia-inducible factor-1α (HIF-1α) is hydroxylated by prolyl hydroxylase domain proteins and bound by pVHL, catalyzing the polyubiquitylation of prolyl hydroxylated HIF-1α for subsequent degradation via the 26S proteasome [16,17]. If pVHL is mutated, as in ccRCC, binding of HIF-1α by pVHL does not occur; the unbound HIF1α is not degraded, but associates with the constitutively stable partner HIF-1β to form an active heterodimeric HIF-1 transcription factor, which binds to hypoxia-responsive elements located in the promoter/enhancer regions of numerous hypoxia-inducible genes. In view of the HIF-1α dependency of CAIX expression, the ubiquitous expression of the G250/CAIX antigen could be explained straightforwardly by nonfunctional VHL gene product in ccRCC (Figure 1).

Elucidation of the molecular pathway of *CAIX* gene expression also readily explained the heterogeneous staining pattern in non-RCC tumors: this is the consequence of local hypoxia, leading to HIF-1α stabilization and subsequent G250/CAIX expression. In fact, G250/CAIX is now regarded as an appropriate substitute hypoxia marker in various tumor types [18,19].

## 3. Clinical Studies with mAbG250

### 3.1. Imaging Studies

The incidental detection of renal lesions has increased up to 50% by improved radiologic imaging, such as contrast enhanced CT and positron emission tomography (PET) with fluorine-18 fluorodeoxyglucose (^18^F-FDG) [21,22]. With increased possibilities for nephron-sparing surgery and the realization that around 20% of these masses are benign tumors, 25% are indolent tumors with limited metastatic potential and 54% represent the more potentially malignant ccRCC, it has become important to differentiate between these entities. However, conventional techniques have difficulties in differentiating between benign and malignant renal lesions. Therefore, surgical interventions are performed that could have been prevented. Consequently, new imaging techniques are needed to improve the differentiation between benign and malignant renal lesions. In view of the ccRCC specificity of mAbG250, multiple studies have addressed its ccRCC targeting capabilities.

Since its discovery, numerous preclinical targeting studies were performed in various mouse models [23–26] with various radionuclides, as well as in *ex vivo* perfusion experiments in tumor-bearing kidneys [27] with mAbG250. Selective and extraordinary high uptake of murine mAbG250 (mG250) in antigen-positive tumor xenografts was observed (e.g., up to more than 100% of the injected dose per gram tumor tissue at the lowest protein doses (up to 1 μg)). The combination of the restricted G250/CAIX expression in normal tissues, homogeneous G250/CAIX expression in RCC and excellent targeting capability in animal models provided a solid basis for the initiation of the clinical evaluation of mG250 in patients to investigate the possibility to use CAIX imaging as a new diagnostic tool.

The first clinical study with mouse mAbG250 (mG250) concerned a phase I presurgical protein dose-escalating study of ^131^I-mG250 conducted to determine tumor uptake and mG250 distribution in patients suspect for RCC [28]. Apart from clear visualization of primary and metastatic (known and occult) RCC at protein doses >2 mg, occult metastases were imaged, immediately demonstrating the diagnostic potential. Levels of mG250 in tumor tissue samples reached levels of up to 0.1% of the injected dose per gram of tumor (%ID/g), these levels being among the highest reported in studies of solid tumors. Additionally, normal tissue uptake, actually limited to the liver, was saturable, encouraging future development of mG250 in RCC. Because histological confirmed CAIX-negative tumors did not image, it was concluded that mAbG250 accumulation was CAIX-specific. Since administration of murine G250 led to the formation of human-anti-mouse-antibodies (HAMA) in all patients, preventing multiple administrations [29], a chimeric variant of G250 (cG250) was constructed (see Table 1).

**Figure 2:**
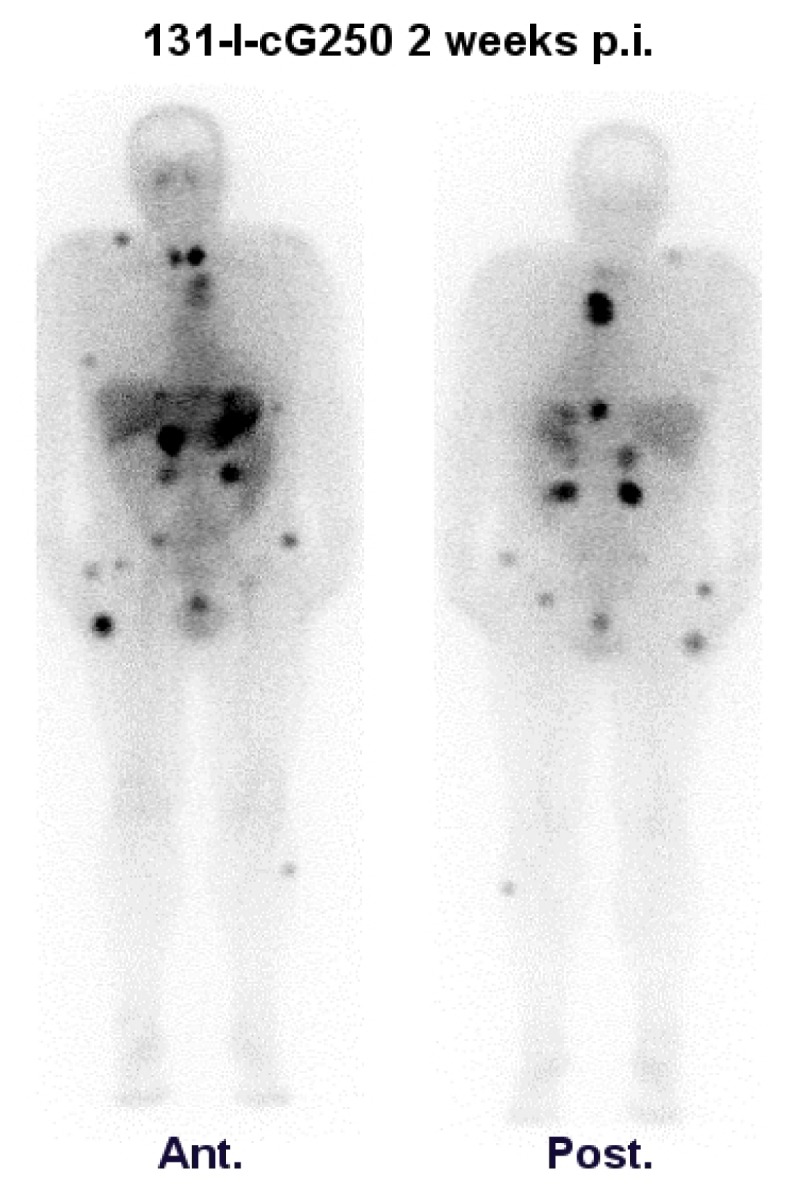
Whole body scan of a patient with multiple RCC metastases two weeks after infusion of 4144 MBq ^131^I-cG250. **Ant**.: Anterior view; **Post**.: Posterior view. Note: thyroid uptake is due to non-specific accumulation, despite attempts to block thyroid uptake with intake of saturated potassium iodine.

Because chimerization might lead to altered pharmacokinetic and pharmacodynamic behavior, a phase I protein dose-escalation trial of ^131^I-cG250 identical to the phase I mG250 protein dose-escalation trial was necessary [30]. All patients with an antigen-positive tumor (*n* = 13) showed excellent targeting of radioactivity to all known tumor sites. Similar to mG250, previously undetected metastatic lesions (brain, bone and soft tissue) were detected. An example of the excellent cG250 uptake is shown in Figure 2. The performance of the chimerized G250 mAb was almost identical to the mouse mAbG250, including the optimal protein dose (5–10 mg) and very high focal uptake (up to 0.52% ID/g). The half-life (t½ β) of cG250 was comparable to mG250 (68.5 h *vs*. 47 h). ^131^I-cG250 uptake in non-tumor tissues remained low. Most importantly, chimerization greatly diminished the immunogenicity of the antibody: in only two of 15 patients, low levels of human anti-chimeric antibody (HACA) were observed [30]. Thus, multiple administrations became feasible.

Although very high uptake levels were observed, locally, cG250 tumor uptake was heterogeneous; this heterogeneity could not be explained by antigen expression alone. No consistent association with necrosis or vasculature was noted [37]. Since highly dynamic vascularization and intratumoral blood flow may contribute to this heterogeneous tumor uptake, the impact of time on tumor uptake of cG250 was studied in a clinical setting. In this dual-label study, ten patients with a clinical diagnosis of primary RCC received two independent consecutive administrations of cG250, separated by four days. Post-surgery, the distribution of both administrations was mapped and analyzed. The study demonstrated that cG250 distribution did not differ between different administrations, indicating that intrinsic tumor factors, such as internalization and local differences in interstitial fluid pressure, played a prominent role in intra-tumoral heterogeneity of antibody distribution [31]). Initially, this explanation was felt to be improbable, because ^131^I-cG250 tumor retention in patients was in the order of weeks, suggesting very low internalization rates [29].

To compare ^131^I-cG250 radioimmunoscintigraphy (RIS) with ^18^F-FDG PET, 20 mRCC patients were scanned using both techniques. Routine imaging modalities, performed before the experimental imaging techniques, revealed 79 metastases in these 20 patients. ^18^F-FDG PET and ^131^I-cG250 scintigraphy revealed 33 previously unknown lesions, of which 32 were PET positive and seven cG250-positive. Remarkably, ^131^I-cG250 RIS detected only 30% (34/112) of documented metastases, whereas with ^18^F-FDG PET, 69% (77/112) were detected [32]. The low percentage of RCC metastases detected by cG250-RIS in this study contrasts with the results of many other studies, where excellent visualization of all known metastases occurred and often new lesions were visualized, which were not seen using conventional imaging techniques. The inferiority of ^131^I-cG250 RIS in detecting metastases might have been due to internalization of the radiolabeled mAb and subsequent excretion of ^131^I by the tumor cells. Internalization and translocation of mAbG250 to the endocytic recycling compartment *in vitro* has been described before [38]. Alternatively, it is possible that many lesions were CAIX-negative, albeit that, in general, approximately 75% of ccRCC metastases are high in CAIX expression [39]. Unfortunately, we were unable to determine the CAIX expression in this trial, since lesions were unavailable.

The dual label clinical trial suggested that cG250 can be internalized by G250 antigen-expressing RCC cells. Indeed, follow-up animal experiments demonstrated that internalization can occur [40] and that accumulation in tumors of cG250 labeled with residualizing radionuclides, such as ^111^In, might be higher than that of non-residualizing ^131^I [25,41]. To investigate this phenomenon in detail in patients with RCC, a dual-label study was performed, with cG250 labeled with the residualizing radionuclide ^111^In and non-residualizing radionuclide ^131^I [33]. Four days post injection, the ^111^In-cG250 images revealed more metastatic lesions (*n* = 47) than ^131^I-cG250 (*n* = 30). Moreover, quantitative analysis of 25 metastases showed higher activities of ^111^In-cG250 than of ^131^I-cG250 in 20 of 25 lesions, thus ^111^In-cG250 outperformed ^131^I-cG250 for visualization of metastatic RCC lesions. This was partly due to the superior gamma camera characteristics of ^111^In, but mainly because higher tumor:blood ratios were obtained.

ImmunoPET—that is, PET scanning that combines the favorable characteristics of PET (higher spatial resolution, three-dimensional imaging and superior quantitative analysis of images) with cG250—seems ideal for RCC imaging. However, the most commonly used positron emitters (^11^C and ^18^F) cannot be combined with the relatively slow pharmacokinetics of intravenously injected radiolabeled mAb (optimal tumor uptake after several days), because of their too-short half-lives (2 min to 1.8 h). On the other hand, the positron emitters, ^89^Zr and ^124^I (half-lives 78 and 100 h, respectively), seem to be good candidates to match these slow kinetics. In the first cG250 immunoPET study, ^124^I-cG250 (185 MBq, 10 mg) was evaluated in 26 patients with suspect renal masses to study whether ccRCC could be recognized unequivocally. In 15/16 patients with histological confirmed ccRCC after surgery, positive images were obtained (one patient received nonreactive antibody, due to technical problems). In addition, all nine non-clear cell renal masses were negative; hence, the sensitivity and specificity of ^124^IG250 PET for ccRCC was 94% and 100%, respectively. The negative (NPV) and positive predictive value (PPV) were 90% and 100%, respectively [34].

This proof of principle study suggested that immunoPET might help in clinical decision-making and might aid in the surgical management of patients with small renal masses scheduled for partial nephrectomy.

To substantiate whether cG250 immunoPET might be helpful in the clinical management of patients with suspect renal masses, a large multicenter phase III trial comparing ^124^I-cG250 immuno PET/computed tomography (CT) (^124^I-Girentuximab/REDECTANE^®^) scanning to contrast enhanced CT (CECT) for the detection of ccRCC was performed. In total, 226 patients scheduled for partial or complete nephrectomy were enrolled in this study [35]. ^124^I-girentuximab was well tolerated, and 195 patients were evaluable. The results of this trial confirmed the high specificity and sensitivity of ^124^I-cG250 for ccRCC. Notably, the average sensitivity and specificity were higher for G250 PET/CT than for CECT (86.2% *vs*. 75.5% and 85.9% *vs*. 46.8%, respectively). The authors concluded that ^124^I-girentuximab PET/CT can accurately and noninvasively identify ccRCC, with potential utility for designing best management approaches for patients with renal masses. One limitation of ^124^I-based immunoPET is the limited availability of ^124^I worldwide, requiring centralized production.

**Figure 3:**
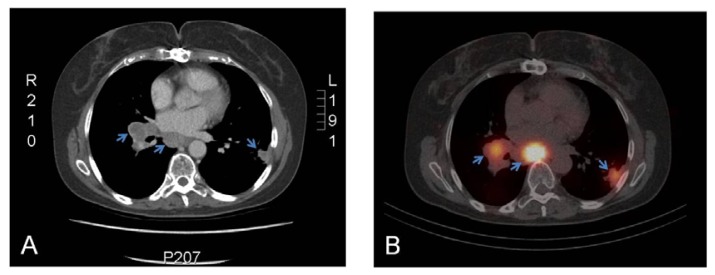
Images of a patient with metastatic RCC. Conventional CT (**A**) and ^111^In-girentuximab immunoSPECT (**B**) images of a patient with metastatic ccRCC. Clear and preferential uptake of the radiolabeled antibody was observed in mediastinal and pleural lesions (arrows). The patient was enrolled in the phase II ^177^Lu-girentuximab RIT trial.

Because girentuximab labeled with the gamma-emitting radionuclide indium ^111^In is easier to produce as an off-the-shelf agent, not requiring centralized production nor specialized equipment, and because dual labeling studies showed superior imaging of ^111^In-cG250 in mRCC [33], we investigated this agent as a potential imaging modality. Similar to ^124^I-girentuximab immunoPET, single-photon emission CT (SPECT) with ^111^In-labeled girentuximab is non-invasive and does not require the use of intravenous contrast agents, which makes it suitable for patients with an impaired renal function. In this study, 29 patients with an incidentaloma of the kidney or having a history of ccRCC with lesions on follow-up imaging suspect for metastases were enrolled [36]. Distinct uptake of ^111^In-girentuximab was seen in 16 of 22 patients presenting with a renal mass (Figure 3). All renal masses proven to be ccRCC after resection (*n* = 15) were detected with ^111^In-girentuximab. In one of the 16 patients, a type 2 papillary RCC with histological proven CAIX expression was targeted with ^111^In-girentuximab. In addition, no targeting was observed in six patients. Histopathological evaluation in 4/6 patients revealed two cases of benign oncocytoma, a chromophobe and a mucinous tubular spindle cell carcinoma subtype tumor. For the two remaining patients, biopsy material was unavailable, but close monitoring with repeated CT scans did not reveal growth of the suspected mass in the follow-up period (>24 months). In this limited group of patients, the PPV of ^111^In-girentuximab imaging for ccRCC was 94%. In addition, seven patients with a history of ccRCC and possible metastatic lesions on follow-up computed tomography scans were imaged with ^111^In-girentuximab. In 4/7 patients, the lesions showed preferential uptake of ^111^In-girentuximab and local or systemic treatment was initiated. In three other cases, no targeting was seen. During follow-up of these three patients, 1/3 showed progression, for which systemic treatment was started. In conclusion, cG250 immunoSPECT either labeled with ^124^I or with ^111^In can be used to detect ccRCC lesions in patients with a primary renal mass and to clarify the nature of lesions suspect for metastases in patients with a history of ccRCC.

### 3.2. Therapy Studies

The therapeutic potential of CAIX targeting with mAbG250 has been studied in numerous clinical trials (see Table 2). Roughly, these can be divided into trials with “naked” antibody alone or in combination with cytokines and radioimmunotherapy trials.

In the first dose escalating radioimmunotherapy (RIT) trial, ^131^I-mG250 was administered to progressive patients with measurable, histological proven ccRCC [29]. In this trial, hepatic toxicity was observed, most likely the result of specific mG250 accumulation in the liver. Indeed, with higher doses, the liver uptake was decreased, suggesting saturation of G250 sites by the antibody. The toxicity was transient and not dose-limiting. As in all RIT studies with radiolabeled antibodies, dose-limiting toxicity (DLT) was hematopoietic. After determining the maximum tolerated dose (MTD) of ^131^I-activity (3330 MBq/m^2^), 15 patients were treated at the MTD level to determine efficacy, but no major responses were noted. However, overall survival of patients treated with ^131^I-mG250 seemed to be increased in comparison with that of historic control patients: 17/33 SD and two minor responses. As mentioned earlier, the development of high HAMA levels in all patients precluded retreatment, and all subsequent trials were carried out with cG250.

Following the protein dose-escalation trial with ^131^I-cG250, which established the most favorable protein dose, a phase I ^131^I-cG250 activity dose escalation was performed to establish DLT similar to the mG250 trial [42]. One major adjustment was the inclusion of an imaging dose (222 MBq of ^131^Ilabeled to 5 mg of cG250), before being allowed to advance to therapeutic dose (1665–2775 MBq of ^131^I- labeled to 5 mg of cG250) to prevent infusion of high-dose ^131^I-cG250 in CAIX-negative patients.

Only those patients showing targeting to tumor (*n* = 8) received the therapeutic infusion of ^131^I-cG250 one week later. Unexpectedly, through the administration of the scout dose, liver toxicity was avoided, most likely because the liver compartment was saturated. Alternatively, hepatic uptake of chimeric mAbG250 is lower than murine mAbG250. At equal doses, liver uptake of mG250 [28] was 2–3-times higher than the liver uptake of cG250 [30]. Dose-limiting toxicity of ^131^I-cG250 was at 2775 MBq ^131^I-cG250/m^2^, significantly lower than DLT observed for the murine version. The MTD was therefore set at 2220 MBq/m^2^. Almost certainly, the extended serum half-life is responsible for the enhanced hematopoietic toxicity, since this leads to extended radiation of the bone marrow compartment.

In one patient, HACA was observed in the serum sample obtained prior to the injection of the radiolabeled antibody, as well as in subsequent serum samples. cG250 was rapidly cleared. The observed HACA was probably due to previous injections with mAbG250. This particular patient had participated four months beforehand in another clinical study and had received two injections of mAbG250, four days apart. Targeting of mAbG250 was observed in his primary tumor at that time. No HACA responses were detected in all other patients.

An antitumor response was observed in 2/8 patients; one SD for 3–6 months and one partial response (PR) >9 months. Both patients were treated at the 2220 MBq/m^2^ dose level. However, quite disappointingly, all other patients showed progression of disease.

This first RIT trial with cG250 clearly showed that increased doses of radioactivity to the tumors were required to achieve more complete and lasting responses. In an effort to increase RIT efficacy, a fractionated dose RIT was performed, based on whole-body radiation absorbed dose [43].

Fractionated RIT is more effective than a single large amount and is associated with a lower toxicity profile in animal models. The primary objective of this trial was to determine the maximum tolerated whole-body radiation-absorbed dose of fractionated ^131^I-cG250, with dose escalation referred to here as the escalation of average whole-body absorbed dose. Fifteen patients with measurable metastatic renal cancer were included. The majority of patients tolerated repeated injections with no change in kinetics, confirming the lack of immunogenicity of the antibody construct. Whole-body and serum kinetics varied significantly between patients, with estimated biologic clearance half-times ranging from 3.2 to 7.5 days for the whole body and from 1.3 to >5 days for serum β–half-life (t½ β). In two of 15 patients, HACA was observed, which lead to a faster serum clearance. Similar to single dose, cG250 RIT, DLT was hematopoietic. In this logistically demanding fractionated administration regimen, sparing of the hematopoietic system was not observed. Moreover, the total dose that could be delivered was low, and efforts along these lines were abandoned.

In view of the minimal clinical response in single doses cG250 RIT, a study was performed with two sequential high-dose (at MTD) ^131^I-cG250 treatments in patients with progressive mRCC [44]. After receiving a scout dose of 185 MBq/m^2^ of ^131^I-cG250 to demonstrate tumor targeting, 29 patients with adequate cG250 uptake received a therapeutic dose of 2220 MBq/m^2 131^I-cG250. In the absence of grade 4 hematological toxicity, patients received a second cycle after three months, consisting of a diagnostic infusion and a second high dose injection of ^131^I-cG250, escalated from 1110 MBq/m^2^ to 1665 MBq/m^2^. The MTD of the second RIT was 1665 MBq/m^2^, with myelotoxicity as DLT. Four patients were excluded from the study, because they developed HACA after the first RIT dose (*n* = 2), after the second scout dose (*n* = 1) or after the second RIT dose. Those patients developed high HACA titers with enhanced clearance of injected mAbG250 (t½ β: 20–27 h). In an additional four patients, detectable HACA titers at low levels developed in the course of the study. In these patients, no enhanced clearance was observed (t½ β: 52–74 h). Of the 16 patients who completed the protocol at both MTDs, none demonstrated an objective response, but five previously progressive patients had stabilization of their disease lasting 3–12 months. The low efficacy was partly attributed to the bulky disease in these end-stage patients, as sufficiently high radiation doses of ^131^I could not be delivered to these large tumor masses. An inverse correlation between the size of metastases and radiation-absorbed dose was observed, and dosimetric analyses showed that therapeutic radiation doses (>50 Gy) were only guided to lesions smaller than 5 g. Therefore, it was suggested that future RIT with cG250 should aim at treatment of small-volume disease or should be used in an adjuvant setting, or other more potent radionuclides should be used [52].

Preclinical animal studies performed with cG250 labeled with more potent radionuclides (^177^Lu, ^90^Y or ^186^Re) for RIT showed that tumor growth was most effectively inhibited by ^177^Lu-cG250, followed by ^90^Y-, ^186^Re- and ^131^I-cG250 [41]. Metabolites labeled with metallic radionuclides, such as ^111^In, ^90^Y and ^177^Lu, are trapped in the lysosomes and residualize after internalization of the mAb–antigen complex by the target cells. Intracellular ^131^I-cG250 is metabolized, and tyrosine-^131^I is rapidly excreted by the tumor cell upon internalization. These RIT studies clearly showed the superiority of ^177^Lu- and ^90^Y-based RIT, in line with other studies. The dual-label study discussed before [33] also supported that trapped radionuclides are superior to the non-trapped iodine. In view of this evidence, subsequent clinical studies have focused on the possibility to use ^90^Y or ^177^Lu in RIT [53].

Recently, the results of a phase I/II trial with ^177^Lu-cG250 were published [45]. This trial was paralleled by a trial with ^90^Y-cG250 at Memorial Sloan Kettering Cancer Centre, New York (clinicaltrials.gov/NCT00199875). In the ^177^Lu-cG250 trial, 23 patients with progressive mRCC with proven ccRCC received a diagnostic dose of ^111^In-cG250 (185 MBq), to establish adequate tumor accumulation followed by a dose of ^177^Lu-cG250 one week post ^111^In-cG250 injection (start dose 1110 MBq/m^2 177^Lu, increments of 370 MBq/m^2^; three patients/dose level). In four patients, elevated HACA levels during treatment were observed. In two patients, these HACA levels precluded administration of a subsequent treatment cycle. In the absence of grade 4 toxicity, patients were eligible to receive a second (13/23) and a third cycle (4/23), at 75% of the dose level of the previous injection. Hematopoietic toxicity was dose-limiting, and MTD was set at 2405 MBq/m2, ^111^In-cG250 images were superimposable on the ^177^Lu-cG250 images, illuminating the predictive value of ^111^In-cG250 for ^177^Lu-cG250 accumulation [45]. In one patient, grade IV toxicity was observed at the 1850 MBq/m^2^ dose level. No significant toxicity was observed in the other patients treated at MTD, also not after the second or third treatment cycle. The majority of patients responded by stabilization of disease. In one patient (1850 MBq/m^2^ dose level), a PR was documented that lasted for nine months. Dosimetric analyses indicated effective uptake after consecutive treatments. Observed hematologic toxicity, especially platelet toxicity, correlated significantly with the administered activity, whole-body absorbed dose and red marrow dose. The tumor-to-red marrow dose ratio was higher for RIT with ^177^Lu-cG250 than for RIT with ^90^Y-cG250, indicating that ^177^Lu has a wider therapeutic window for RIT with cG250 than ^90^Y. The authors concluded that in patients with metastasized renal cell carcinoma, higher radiation doses can be guided to the tumors with ^177^Lu-cG250 than with ^90^Y-cG250 [54]. The authors concluded that RIT with ^177^Lu-cG250, targeting CAIX, may stabilize previously progressive metastatic ccRCC.

### 3.3. Therapeutic Studies with Unmodified cG250

Antibodies have the capacity to lyse cells by complement activation or by antibody-dependent cellular cytotoxicity (ADCC). *In vitro* studies established that cG250 could initiate cell lysis through ADCC of CAIX-positive cells [55,56]. Also, significant tumor growth reduction was noted when mice bearing human RCC xenografts were treated with naked mAbG250 [49]. Based on these results, a phase 1 study with escalating doses of 5–50 mg/m^2^ of cG250 (Girentuximab/RENCAREX^®^), with weekly infusions for six6 weeks, was initiated. Treatment up to the highest dose was safe and well tolerated. Of the 11 mRCC patients treated, one patient showed a CR and nine patients had SD after one treatment cycle [46].

In the subsequent phase 2 study, 36 patients with advanced RCC were included, all received 50 mg of cG250 weekly for 12 weeks. Before treatment, 80% of patients were progressive. After one treatment cycle, 28% of previously progressive patients had SD for at least six months, suggesting a clinical benefit [47]. During follow-up, one CR and one PR were noted, which lasted >1 year. The median survival of 15 months with 41% of the 32 evaluable patients still alive after two years suggested that cG250 might be able to modulate the natural course of mRCC. One group of patients received extended treatment (an additional eight weeks of treatment). These patients showed a median survival of 39 months, compared to 10 months in the discontinued group. Patients receiving extended treatment with cG250 showed a significantly longer survival rate than the nonresponsive patients (70% *vs*. 26%). The levels of mAbG250-mediated ADCC differed between the patients: 42% of the patients showed moderate ADCC (5%–25%), whereas in 33% of patients, no ADCC was demonstrated. There was no clear correlation between the *in vitro* levels of cytotoxicity and the clinical responses. In addition, no correlation between the proportion of NK cells and the level of mAbG250-mediated ADCC was observed. In this non-randomized setting, it has been difficult to evaluate the true effect of cG250 treatment on the disease course of patients, as the natural disease course of mRCC is highly variable and periods with SD and/or PR can occur, even in the absence of treatment.

Based on these results, an adjuvant double-blind, placebo controlled phase 3 trial was started (ARISER, Adjuvant RENCAREX^®^ Immunotherapy Phase III trial to study efficacy in non-metastatic RCC), aiming at reducing the recurrence of disease in nephrectomized RCC patients who have a high risk of relapse (http://www.wilex.de/portfolio-english/rencarex/phase-III-ariser/) [48]. The trial recruited 864 patients with prior nephrectomy of primary ccRCC; patients received a once-weekly infusion of RENCAREX^®^ or placebo for 24 weeks. Those patients receiving the active drug received a loading dose of 50 mg in week 1 and weekly doses of 20 mg during weeks 2–24.

Unfortunately, the trial did not meet its primary endpoint. The analysis showed no improvement in median DFS (approximately 72 months) following RENCAREX^®^ treatment compared with placebo. However, preliminary results of a retrospective subanalysis appear to indicate that with increasing CAIX expression in tumor tissue, as quantified by a CAIX score, the treatment was more effective; DFS showed a clinically and statistically significant improvement in the patient population with a high CAIX level treated with cG250 compared to both placebo and patients with a low CAIX score (press release Wilex, 26 February, 2013). No other data are available at present. Therefore, an immunotherapy for ccRCC in the adjuvant setting might still be an option in a highly defined subpopulation.

Since interleukin-2 (IL-2) has been known to enhance ADCC of mAbs, the combination of this cytokine with cG250 was evaluated. *In vitro* studies had demonstrated that cG250 ADCC was increased when cells from IL-2-treated patients were used, suggesting that the combination of cG250 with IL-2 might be superior to cG250 alone [55]. In a phase 2 trial, 35 patients with progressive mRCC received weekly intravenous infusions of 50 mg of cG250 and daily subcutaneous low-dose IL-2 for 11 weeks. Treatment was well tolerated with little toxicity, attributable to IL-2. Clinical benefit was noted in eight of 35 patients (23%), with one long-lasting PR (>95 weeks), six long-lasting SD (>24 weeks) and a mean survival of 24 months, with 45% of the 30 evaluable patients still alive after two years. The extended treatment group (an additional six weeks of treatment) showed a median survival of 41 months, compared with 13 months in the non-response group. Patients receiving extended treatment showed a significantly longer survival rate than the non-response patients (55% *vs*. 25%). The increased survival (as compared to historic controls) was deemed to be cG250-related and not related to the IL-2, as a six-fold decrease of the normal IL-2 dose was used [50]. The favorable outcome might have been due to a synergistic effect of cG250 and IL-2, as was observed in the *in vitro* studies.

The number of effector cells (CD3−/CD16+/CD56+) increased during treatment, but lytic capacity per cell did not increase and ADCC and clinical outcome did not correlate. Similar results were observed in the study performed by Davis *et al*. [49]. Yet again, because patients were not randomized, it is difficult to judge the value of this observation. A randomized trial is needed to determine the true effect of the IL-2/cG250 treatment.

Based on *in vitro* observations that with the addition of Interferons (IFNs), G250/CAIX expression was upregulated and that with Interferon gamma (IFN-γ), ADCC of cG250 was enhanced, the effect of cG250 combined with IFN-α was studied [56,57].

In a multicenter, open-label, prospective, single-arm phase I/II trial study, cG250 in combination with IFN-α 2a was studied in a total of 32 patients with stage IV progressive RCC [51]. Patients received 20 mg cG250 weekly for three months, combined with IFN-α 2a, three million international unit (MIU), three times per week, subcutaneously. Twenty six of 31 patients were evaluable for response to treatment. Two patients showed a PR and 14 patients SD in week 16. One patient experienced a PR for at least eight months, and nine patients had long durable disease stabilization (≥24 weeks). Clinical benefit was obtained in 42% (11/26) of the patients. The overall median survival was 30 months for the 31 patients treated with WX-G250 and IFNα, with 57% of patients still alive after two years. The patients receiving extended treatment showed a median survival of 45 months compared with 10 months in the non-extended group. Patients receiving extended treatment with cG250 showed a significantly longer survival rate than the non-response patients (79% *vs.* 30%).

## 4. Future Prospects

Clinical studies have now firmly established that cG250 adequately targets ccRCC. However, to prove that cG250 imaging can be used to guide clinical management, more evidence is needed. Thus far, patients for whom surgery was part of their clinical management have been studied, but the question whether watchful waiting can be applied in patients in whom no cG250 targeting is seen remains to be answered. As suggested by Divgi *et al*. [35], the role of cG250 imaging in influencing outcome would perhaps be best assessed in a clinical trial carried out in patients with small renal mass tumors and associated comorbidities.

Another issue that needs further study is choice of radionuclide for imaging purposes. As discussed, ^111^In-cG250 SPECT may provide the same information as ^124^I-cG250 PET, but with the advantage that this can be produced as an off-the-shelf product that can be used on site. Alternatively, other radionuclides, such as ^89^Zr, might provide a useful alternative: it is similar with respect to its chemical characteristics, but is PET imageable. Although the adjuvant study in a group of high-risk patients did not reach it primary end-point, it is noteworthy to mention that in a subgroup analysis, patients with high CAIX expression as determined by IHC showed lower recurrence than patient negative or low in CAIX expression levels. Biomarker studies have demonstrated that CAIX staining correlates with survival, as well as with response to high dose IL-2 [58,59]. A cutoff of 85% CAIX staining provided the most accurate prediction of survival. Low CAIX (≤85%) staining was an independent poor prognostic factor for survival for patients with mRCC. For patients with nonmetastatic RCC and at high risk for progression, low CAIX predicted a worse outcome similar to patients with metastatic disease. Intriguingly, CAIX expression correlated with response to high dose IL-2: survival >5 years was only seen in high CAIX expressers [60,61]. It is tempting to speculate that high-dose IL-2 treatment leads to expansion of CAIX-specific CTL, but characterization of 18 different TIL cultures suggested that anti-G250 reactivity is rare [13]. Patient stratification based on CAIX expression might lead to a (adjuvant) treatment strategy similar to, e.g., trastuzumab treatment of patients with breast cancer, where a very high correlation exists between HER2 expression and the success of trastuzumab, which targets HER2 [62].

In summary, mAbG250 has shown remarkable targeting ability, and the main value of the antibody at present appears to be as diagnostic and, also, as a delivery vehicle for RIT.

cG250 PET imaging holds great promise for the future, both in detecting localized and advanced disease, albeit that the radioisotope to use is still under investigation. In the near future, a clinical trial with PET tracer ^89^Zr-girentuximab will be initiated in our center, which will provide additional information about the use of girentuximab-based immunoPET in ccRCC.

Clinical trials with unmodified cG250 suggest that treatment with cG250 can influence the disease course in mRCC patients. However, to validate whether cG250 treatment is of value, large, randomized trials are needed.

Besides the usefulness in radioimmunodetection, girentuximab is a potent carrier for RIT in ccRCC. However, there are still several hurdles to overcome before girentuximab-based RIT can be implemented as a standard treatment. As previously mentioned, it is not clear which patients benefit most from RIT. Past results indicate that RIT is mainly suitable for treatment of small-volume disease or, possibly, as adjuvant treatment in selected cases, and more evidence regarding this topic is expected from the ongoing clinical trials with ^90^Y and ^177^Lu-labeled girentuximab in the upcoming years. Besides better patient selection in the future, advances in dosimetric analysis will presumably contribute to the improvement of RIT, as the trade-off between efficacy and toxicity can be better tailored to the individual patient. Lastly, an important deficit in our current knowledge is how to optimally combine girentuximab-based RIT with the current standard of care in metastatic ccRCC.

*CAIX* has also been used as a target in gene therapy studies with modified autologous T-cells [63]. In these studies, patients with advanced RCC were infused with escalating doses of T-cells genetically retargeted with a chimeric antibody receptor (CAR) directed towards carbonic anhydrase IX. Thus, the antigen-specific variable regions of mAbG250 (targeting to RCC) were linked to T-cell receptor signaling chains, leading to *CAIX* targeting in an MHC-independent context. Liver toxicity was observed at the lowest cell dose, illustrating the potency of the modified T-cells. The liver toxicity could be prevented by pre-dosing with cG250 before administering modified T-cells [64]. Given that similar to mAb studies, the observed “on”-target toxicity could be prevented by blocking antigenic sites in off-tumor organs, higher T-cell doses might be possible. Although this is a very intriguing approach, patient recruitment has been very slow, and the final results are awaited.

Finally, the clinical management of mRCC has changed significantly over the last few years. Implementation of various tyrosine kinase inhibitors and mTOR inhibitors has led to improved progression-free survival. However, therapy resistance is a major issue, and these therapies are directed against the tumor vasculature and not against the tumor cells. Combination of TKI and cG250 might be beneficial: they attack different targets, and the combination might lead to synergistic effects. In a preclinical study, the biodistribution of cG250 was determined in TKI treated mice. TKI are known anti-angiogenic drugs, and thus, the accessibility of tumor cells is likely to be altered. In nude mice bearing human RCC xenografts treated orally with sunitinib, vandetanib or sorafenib, tumor uptake of cG250 decreased dramatically, and vascular density decreased considerably, as judged by various markers [65]. This is comparable to the TKI effects on tumors in patients: large central necrotic areas can develop in tumors when patients are treated with TKI. When treatment was stopped, robust neovascularization, mainly at the tumor periphery, became apparent. Consequently, cG250 uptake recovered, albeit that cG250 uptake appeared to be restricted to the tumor periphery, where vigorous neovascularization was visible. This animal study demonstrated that simultaneous administration of TKI and mAb cG250 is unlikely to improve therapy outcome. This was also demonstrated in patients; a markedly decreased uptake of ^111^In-girentuximab after treatment with TKI sorafenib was observed [66]. Data from this study suggest that the effect of girentuximab-based RIT would be severely hampered if given during TKI treatment. However, the observation that shortly after discontinuation of TKI treatment, mAb accumulation was restored, suggested that sequential treatment strategies might be useful [65]. Because cG250 and TKI appear to be feasible in sequence only, studies were designed to determine the optimal interval between TKI treatment and cG250 administration. Biodistribution studies in two different animal models demonstrated that a 3D time interval was sufficient to reach optimal antibody accumulation (manuscript in preparation). Because TKI are also given in cycles in patients (four week treatment followed by two weeks off treatment), it appears that, indeed, combination of Sunitinib with mAb cG250 is feasible in mRCC patients, but the optimal treatment schedule needs to be determined.

Anti-CAIX antibodies that combine inhibition of the enzymatic activity of CAIX with ADCC and/or CDC might be more potent than mAbG250, which does not inhibit the enzymatic activity of CAIX. Recently, a CAIX-specific antibody with an inhibitory effect on the carbonic anhydrase activity was described [67]. Up to 76% of CAIX activity was inhibited with the full-length IgG antibody MSC8. The authors speculated that combining target specificity with enzymatic inhibition in one antibody molecule may have an additive effect on reducing tumor growth. This interesting hypothesis will need to be substantiated.

## 5. Conclusion

In summary, mAbG250 has shown outstanding targeting ability, and cG250 PET imaging holds great promise for the future, both in detecting localized and advanced disease, albeit that the most favorable radioisotope still needs to be determined.

Confirmation of the results obtained in the non-randomized clinical trials with unmodified cG250 is needed to substantiate the value of cG250 treatment in mRCC. Girentuximab-based RIT hold promise for treatment of patients with small-volume disease or, possibly, as adjuvant treatment in selected cases, and ongoing clinical trials in the upcoming years should provide evidence for this assumption. Lastly, whether combination of girentuximab-based RIT with the current TKI is possible needs to be established.

## Figures and Tables

**Table 1:** Overview of Imaging studies with mAbG250 in RCC patients.

Ref.	Year	Agent	Number of patients	Patients	Outcome #	Remarks
Oosterwijk *et al*. [28]	1993	^131^I-mG250	15	Primary RCC	12/12 pts	Phase I, dose escalation
Steffens *et al*. [30]	1997	^131^I-cG250	16	Primary RCC	13/13 pts	Phase I, dose escalation
Steffens *et al*. [31]	1999	^131^I-cG250 & ^111^In-cG250	10	Primary RCC	10/10 pts	Dual label study
Brouwers *et al*. [32]	2002	^131^I-cG250 *vs*. ^18^F-FDG	20	M + RCC	^131^I-cG250: 34/112 lesions ^18^F-FDG: 77/112 lesions	Comparative intrapatient study
Brouwers *et al*. [33]	2003	^131^I-cG250 & ^111^In-cG250	5	M + RCC	^111^In-cG250: 47 lesions ^131^I-cG250: 30 lesions	Comparative intrapatient study
Divgi *et al*. [34]	2007	^124^I-cG250	26	Primary RCC	15/16 ccRCC imaged	Prospective cG250-immunoPET
Divgi *et al*. [35]	2013	^124^I-cG250	226	Primary RCC	124/143 ccRCC imaged (sens. & spec. 86%)	Phase III, REDECT trial
Muselaers *et al*. [36]	2013	^111^In-cG250	29	Primary RCC	15/16 ccRCC imaged	^111^In-cG250 immunoSPECT

# outcome refers to diagnostic accuracy, *i.e*., number of positive images over total number of images mG250: mouse monoclonal antibody G250; cG250: chimeric monoclonal antibody G250; ccRCC: clear-cell renal cell carcinoma; ^18^F-FDG: fluorine-18 fluorodeoxyglucose; M + RCC: metastatic renal cell carcinoma; PET = positron emission tomography; SPECT: single-photon emission CT; sens.: sensitivity; spec.: specificity.

**Table 2:** Overview of Therapy studies with mAbG250 in RCC patients.

Ref.	Year	Agent	Number of patients	Patients	Response	Duration Response	Remarks
Divgi *et al*. [29]	1998	^131^I-mG250	33	M + RCC	17 SD; 16 PD	2–3 mo	Phase I/II
Steffens *et al*. [42]	1999	^131^I-cG250	12	M + RCC	1 PR; 1 SD; 10 PD	9+; 3–6 mo	Phase I Activity dose
Divgi *et al*. [43]	2004	^131^I-cG250	15	M + RCC	7 SD; 8 PD	2–11 mo	Phase I fractionated dose
Brouwers *et al*. [44]	2005	^131^I-cG250 Two doses	27	M + RCC	5 SD; 22 PD	3–12 mo	Phase I two high doses
Stillebroer *et al*. [45]	2012	^177^Lu-cG250 Multiple doses	23	M + RCC	1 PR; 17 SD	9+; 3+ mo	Phase I dose escalation
Davis *et al*. [46]	2007	cG250	12	M + RCC	1 CR; 8 SD; 3 PD	6–66 wk	Phase I
Bleumer *et al*. [47]	2004	cG250	36	M + RCC	1 CR; 1 PR; 8 SD; 26 PD	1–20+ wk	Phase II
ARISER [48]		cG250	864	High risk, after nephrectomy	No benefit *		Phase III
Davis *et al*. [49]	2007	cG250 + IL-2	9	M + RCC	2 SD; 7 PD	6, 12 wk	Phase I
Bleumer *et al*. [50]	2006	cG250 + IL-2	35	M + RCC	1 PR; 7 SD; 27 PD	95+; 24+ wk	Phase II
Siebels *et al*. [51]	2011	cG250 + IFN-2α	31	M + RCC	1 CR; 9 SD	17+; 24+ wk	Phase II

* No benefit for whole population, high CAIX expression correlated with risk of recurrence reduction; mG250: mouse monoclonal antibody G250; cG250: chimeric monoclonal antibody G250; ccRCC: clear-cell renal cell carcinoma; M + RCC: metastatic renal cell carcinoma; IL-2: interleukin-2; IFN: interferon; CR: complete response; PR: partial response; SD: stable disease; PD: progressive disease; mo: months; wk: weeks.
